# Supplementary material for: Resolving the full spectrum of human genome variation using Linked-Reads
Source: Genome Res. 2019 Apr;29(4):635–45. doi: 10.1101/gr.234443.118 (PMC6442396; doi:10.1101/gr.234443.118)
Supplement: Supplemental Material [file supp_gr.234443.118_Supplemental_longranger-2.2.zip › longranger-2.2.tar.bz2!/lr2.2-public/lib/stan/cmdstan/stan/doc/api/html/math_2matrix_8hpp_source.html]

Stan: src/stan/math/matrix.hpp Source File


|  |  |
| --- | --- |
|  | Stan  2.5.0  probability, sampling & optimization |


- Main Page
- Related Pages
- Namespaces
- Classes
- Files

- File List
- File Members

All Classes Namespaces Files Functions Variables Typedefs Enumerations Enumerator Friends Macros Pages

- src
- stan
- math

matrix.hpp

Go to the documentation of this file.

1 #ifndef STAN\_\_MATH\_\_MATRIX\_HPP

2 #define STAN\_\_MATH\_\_MATRIX\_HPP

3

4 #include <stan/math/matrix/Eigen.hpp>

5 #include <stan/math/matrix/accumulator.hpp>

6 #include <stan/math/matrix/add.hpp>

7 #include <stan/math/matrix/append\_row.hpp>

8 #include <stan/math/matrix/array\_builder.hpp>

9 #include <stan/math/matrix/assign.hpp>

10 #include <stan/math/matrix/block.hpp>

11 #include <stan/math/matrix/append\_col.hpp>

12 #include <stan/math/matrix/cholesky\_decompose.hpp>

13 #include <stan/math/matrix/col.hpp>

14 #include <stan/math/matrix/cols.hpp>

15 #include <stan/math/matrix/columns\_dot\_product.hpp>

16 #include <stan/math/matrix/columns\_dot\_self.hpp>

17 #include <stan/math/matrix/common\_type.hpp>

18 #include <stan/math/matrix/containers\_conversion.hpp>

19 #include <stan/math/matrix/crossprod.hpp>

20 #include <stan/math/matrix/cumulative\_sum.hpp>

21 #include <stan/math/matrix/determinant.hpp>

22 #include <stan/math/matrix/diag\_matrix.hpp>

23 #include <stan/math/matrix/diag\_post\_multiply.hpp>

24 #include <stan/math/matrix/diag\_pre\_multiply.hpp>

25 #include <stan/math/matrix/diagonal.hpp>

26 #include <stan/math/matrix/dims.hpp>

27 #include <stan/math/matrix/distance.hpp>

28 #include <stan/math/matrix/divide.hpp>

29 #include <stan/math/matrix/dot\_product.hpp>

30 #include <stan/math/matrix/dot\_self.hpp>

31 #include <stan/math/matrix/eigenvalues\_sym.hpp>

32 #include <stan/math/matrix/eigenvectors\_sym.hpp>

33 #include <stan/math/matrix/elt\_divide.hpp>

34 #include <stan/math/matrix/elt\_multiply.hpp>

35 #include <stan/math/matrix/exp.hpp>

36 #include <stan/math/matrix/fill.hpp>

37 #include <stan/math/matrix/get\_lp.hpp>

38 #include <stan/math/matrix/get\_base1.hpp>

39 #include <stan/math/matrix/get\_base1\_lhs.hpp>

40 #include <stan/math/matrix/head.hpp>

41 #include <stan/math/matrix/initialize.hpp>

42 #include <stan/math/matrix/inverse.hpp>

43 #include <stan/math/matrix/inverse\_spd.hpp>

44 #include <stan/math/matrix/log.hpp>

45 #include <stan/math/matrix/log\_determinant.hpp>

46 #include <stan/math/matrix/log\_determinant\_ldlt.hpp>

47 #include <stan/math/matrix/log\_determinant\_spd.hpp>

48 #include <stan/math/matrix/log\_softmax.hpp>

49 #include <stan/math/matrix/log\_sum\_exp.hpp>

50 #include <stan/math/matrix/max.hpp>

51 #include <stan/math/matrix/mdivide\_left.hpp>

52 #include <stan/math/matrix/mdivide\_left\_ldlt.hpp>

53 #include <stan/math/matrix/mdivide\_left\_tri.hpp>

54 #include <stan/math/matrix/mdivide\_left\_tri\_low.hpp>

55 #include <stan/math/matrix/mdivide\_right.hpp>

56 #include <stan/math/matrix/mdivide\_right\_ldlt.hpp>

57 #include <stan/math/matrix/mdivide\_right\_tri.hpp>

58 #include <stan/math/matrix/mdivide\_right\_tri\_low.hpp>

59 #include <stan/math/matrix/mean.hpp>

60 #include <stan/math/matrix/meta.hpp>

61 #include <stan/math/matrix/min.hpp>

62 #include <stan/math/matrix/minus.hpp>

63 #include <stan/math/matrix/multiply.hpp>

64 #include <stan/math/matrix/multiply\_lower\_tri\_self\_transpose.hpp>

65 #include <stan/math/matrix/num\_elements.hpp>

66 #include <stan/math/matrix/prod.hpp>

67 #include <stan/math/matrix/promote\_common.hpp>

68 #include <stan/math/matrix/promoter.hpp>

69 #include <stan/math/matrix/promote\_scalar.hpp>

70 #include <stan/math/matrix/promote\_scalar\_type.hpp>

71 #include <stan/math/matrix/qr\_Q.hpp>

72 #include <stan/math/matrix/qr\_R.hpp>

73 #include <stan/math/matrix/quad\_form.hpp>

74 #include <stan/math/matrix/quad\_form\_diag.hpp>

75 #include <stan/math/matrix/rank.hpp>

76 #include <stan/math/matrix/resize.hpp>

77 #include <stan/math/matrix/row.hpp>

78 #include <stan/math/matrix/rows.hpp>

79 #include <stan/math/matrix/rows\_dot\_product.hpp>

80 #include <stan/math/matrix/rows\_dot\_self.hpp>

81 #include <stan/math/matrix/sd.hpp>

82 #include <stan/math/matrix/segment.hpp>

83 #include <stan/math/matrix/singular\_values.hpp>

84 #include <stan/math/matrix/size.hpp>

85 #include <stan/math/matrix/softmax.hpp>

86 #include <stan/math/matrix/sort.hpp>

87 #include <stan/math/matrix/sort\_indices.hpp>

88 #include <stan/math/matrix/squared\_distance.hpp>

89 #include <stan/math/matrix/stan\_print.hpp>

90 #include <stan/math/matrix/sub\_col.hpp>

91 #include <stan/math/matrix/sub\_row.hpp>

92 #include <stan/math/matrix/subtract.hpp>

93 #include <stan/math/matrix/sum.hpp>

94 #include <stan/math/matrix/tail.hpp>

95 #include <stan/math/matrix/tcrossprod.hpp>

96 #include <stan/math/matrix/trace.hpp>

97 #include <stan/math/matrix/trace\_gen\_inv\_quad\_form\_ldlt.hpp>

98 #include <stan/math/matrix/trace\_gen\_quad\_form.hpp>

99 #include <stan/math/matrix/trace\_inv\_quad\_form\_ldlt.hpp>

100 #include <stan/math/matrix/trace\_quad\_form.hpp>

101 #include <stan/math/matrix/transpose.hpp>

102 #include <stan/math/matrix/typedefs.hpp>

103 #include <stan/math/matrix/variance.hpp>

104

105 #endif

106

columns\_dot\_self.hpp

mdivide\_left\_tri\_low.hpp

mdivide\_left\_ldlt.hpp

diagonal.hpp

cols.hpp

mdivide\_right.hpp

divide.hpp

get\_base1\_lhs.hpp

size.hpp

num\_elements.hpp

prod.hpp

typedefs.hpp

eigenvectors\_sym.hpp

sd.hpp

multiply\_lower\_tri\_self\_transpose.hpp

columns\_dot\_product.hpp

tail.hpp

quad\_form.hpp

promote\_scalar\_type.hpp

promote\_common.hpp

trace\_quad\_form.hpp

elt\_divide.hpp

add.hpp

segment.hpp

promoter.hpp

sort\_indices.hpp

crossprod.hpp

log.hpp

diag\_matrix.hpp

resize.hpp

variance.hpp

log\_determinant\_spd.hpp

mdivide\_right\_tri.hpp

sub\_col.hpp

transpose.hpp

trace.hpp

dims.hpp

dot\_self.hpp

mdivide\_right\_ldlt.hpp

containers\_conversion.hpp

qr\_R.hpp

cumulative\_sum.hpp

initialize.hpp

promote\_scalar.hpp

mdivide\_left\_tri.hpp

sort.hpp

get\_base1.hpp

rows\_dot\_product.hpp

log\_determinant\_ldlt.hpp

trace\_gen\_inv\_quad\_form\_ldlt.hpp

quad\_form\_diag.hpp

determinant.hpp

mean.hpp

diag\_pre\_multiply.hpp

log\_determinant.hpp

head.hpp

fill.hpp

col.hpp

inverse.hpp

rank.hpp

softmax.hpp

Eigen.hpp

log\_softmax.hpp

dot\_product.hpp

minus.hpp

row.hpp

mdivide\_right\_tri\_low.hpp

accumulator.hpp

rows\_dot\_self.hpp

trace\_inv\_quad\_form\_ldlt.hpp

block.hpp

get\_lp.hpp

mdivide\_left.hpp

common\_type.hpp

min.hpp

trace\_gen\_quad\_form.hpp

append\_row.hpp

array\_builder.hpp

elt\_multiply.hpp

max.hpp

log\_sum\_exp.hpp

tcrossprod.hpp

squared\_distance.hpp

diag\_post\_multiply.hpp

meta.hpp

assign.hpp

subtract.hpp

sum.hpp

stan\_print.hpp

qr\_Q.hpp

multiply.hpp

sub\_row.hpp

eigenvalues\_sym.hpp

distance.hpp

inverse\_spd.hpp

cholesky\_decompose.hpp

exp.hpp

rows.hpp

append\_col.hpp

singular\_values.hpp


---

[ Stan Home Page ]

*© 2011–2014,
Stan Development Team.*
